# DNA methylation markers in the diagnosis and prognosis of common leukemias

**DOI:** 10.1038/s41392-019-0090-5

**Published:** 2020-01-10

**Authors:** Hua Jiang, Zhiying Ou, Yingyi He, Meixing Yu, Shaoqing Wu, Gen Li, Jie Zhu, Ru Zhang, Jiayi Wang, Lianghong Zheng, Xiaohong Zhang, Wenge Hao, Liya He, Xiaoqiong Gu, Qingli Quan, Edward Zhang, Huiyan Luo, Wei Wei, Zhihuan Li, Guangxi Zang, Charlotte Zhang, Tina Poon, Daniel Zhang, Ian Ziyar, Run-ze Zhang, Oulan Li, Linhai Cheng, Taylor Shimizu, Xinping Cui, Jian-kang Zhu, Xin Sun, Kang Zhang

**Affiliations:** 1 Guangzhou Women and Children’s Medical Center, Guangzhou Medical University, Guangzhou, 510623 China; 2 Guangzhou Regenerative Medicine and Health Guangdong Laboratory, Guangzhou, 510005 China; 3 State Key Laboratory of Oncology, Sun Yat-sen University Cancer Center, Guangzhou, 510060 China; 4 Department of Statistics and Institute for Integrative Genome Biology, University of California Riverside, Riverside, CA 92521 USA; 5 Shanghai Center for Plant Stress Biology, Shanghai Institute for Biological Sciences, Chinese Academy of Sciences, Shanghai, 210602 China; 6 Faculty of Medicine, Macau University of Science and Technology, Taipa, Macau China

**Keywords:** Haematological cancer, Prognostic markers

## Abstract

The ability to identify a specific type of leukemia using minimally invasive biopsies holds great promise to improve the diagnosis, treatment selection, and prognosis prediction of patients. Using genome-wide methylation profiling and machine learning methods, we investigated the utility of CpG methylation status to differentiate blood from patients with acute lymphocytic leukemia (ALL) or acute myelogenous leukemia (AML) from normal blood. We established a CpG methylation panel that can distinguish ALL and AML blood from normal blood as well as ALL blood from AML blood with high sensitivity and specificity. We then developed a methylation-based survival classifier with 23 CpGs for ALL and 20 CpGs for AML that could successfully divide patients into high-risk and low-risk groups, with significant differences in clinical outcome in each leukemia type. Together, these findings demonstrate that methylation profiles can be highly sensitive and specific in the accurate diagnosis of ALL and AML, with implications for the prediction of prognosis and treatment selection.

## Introduction

Acute lymphocytic leukemia (ALL) and acute myelogenous leukemia (AML), two common types of human acute leukemia, arise from hematopoietic progenitors of lymphoid or myeloid lineage or from hematopoietic stem cells. The diagnosis of leukemia based on pathological and molecular subtype as well as other histological markers is currently the gold standard for the selection of proper treatment and prognosis stratification.^1–3^ Immunological and molecular-based classifications are also used in the treatment decision-making process for ALL or AML. However, they still lack accuracy, especially in prognosis and survival predictions.

Epigenetic changes such as chromatin modification, microRNA expression changes, and DNA methylation changes have been reported extensively in cancer studies.^4^ The methylation pattern of CpG sites is an epigenetic regulator of gene expression.^5,6^ Extensive alterations in DNA methylation have been noted in almost all cancer types, causing changes in gene expression that promote oncogenesis.^5,7,8^ Both epigenetic and somatic mutations have promise for improving the characterization of malignancy to predict treatment response and prognosis.^7,9–11^ Particular changes in methylation profiles are postulated to be reproducibly found in specific cancer types. In contrast, somatic mutations, with some notable exceptions, typically show neither specificity nor sensitivity for a particular cancer type. Even within commonly mutated genes, individual mutations may be found across tens or hundreds of kilobases, limiting the utility of targeted sequencing of these molecular markers.^12,13^

Methods for DNA methylation evaluation can be classified into enzyme-based, anti-methylcytosine antibody-based, and bisulfate treatment-based approaches.^14^ Although each approach provides specific advantages over the others, the bisulfate treatment-based method has been the most widely utilized method due to its reproducibility and single base-pair resolution and the existence of particulate padlock primer-based bisulfate sequencing.^15,16^ Compared to other bisulfate treatment-based methods, the padlock-based method is more cost-effective, methylation position-specific, and flexible to modification; therefore, it has been commonly utilized for single-base-pair-resolution analysis.^17^ In our study, a padlock probe set was generated from 729 CpG markers that showed differential methylation values in many cancer types when compared to the corresponding normal tissues.^18^

Thus, to explore the utility of methylation patterns in differentiating leukemic cancers and improving prognosis, we analyzed the whole-genome methylation profiles of blood samples from patients with ALL and AML and healthy controls. We also used methylation patterns to predict survival in these patients. These markers not only outperformed present-day methods in their high sensitivity and specificity for diagnosis but also demonstrated the effect of stratifying patients with different prognoses.

## Results

### Characteristics of patients

Clinical characteristics and molecular profiles, including methylation data for our study cohort, were obtained for 194 AML patients, 136 ALL patients, and 754 healthy individuals. The clinical characteristics of the AML and ALL patients in the study cohorts and healthy controls are listed in Table 1.

**Table 1:** Clinical characteristics.

Characteristic	AML	ALL	Normal
Total (*n*)	194	136	754
Gender			
Femal-no. (%)	90 (46)	42 (31)	401 (53)
Male-no. (%)	104 (54)	94 (69)	353 (47)
Age at diagnosis (year)			
Median	55	5	63
Range	18–88	1–13	19–101
White race-no/total no. (%)			
White	176 (91)	0	504 (67)
Asian	2 (1)	136 (100)	7 (1)
Other	16 (8)	0	243 (32)
White cell count at diagnosis (×10^9^/L)			
Mean	37.94 ± 30.72	8.15 ± 5.78	NA
Median	17	5	NA
FAB subtype — no. (%)			
AML with minimal maturation: M0	19 (10)	NA	NA
AML without maturation: M1	42 (22)	NA	NA
AML with maturation: M2	43 (22)	NA	NA
Acute promyelocytic leukemia: M3	19 (10)	NA	NA
Acute myelomonocytic leukemia: M4	41 (21)	NA	NA
Acute monoblastic or monocytic leukemia: M5	22 (11)	NA	NA
Acute erythroid leukemia: M6	3 (1.5)	NA	NA
Acute megakaryoblastic leukemia: M7	3 (1.5)	NA	NA
ALL-L1	NA	74 (55)	NA
ALL-L2	NA	37 (27)	NA
ALL-L3	NA	14 (10)	NA
Other subtype	2 (1)	11 (8)	NA
Cytogenetic risk group-no (%)			
Favorable (Low risk)	36 (19)	19 (14)	NA
Intermediate (Standard risk)	110 (57)	64 (47)	NA
Unfavorable (High/Very high risk)	43 (22)	39 (29)	NA
Missing data	3 (2)	14 (10)	NA

ALL-L1: Small cells with homogeneous nuclear chromatin, a regular nuclear shape, small or no nucleoli, scanty cytoplasm, and mild to moderate

ALL-L2: Large, heterogeneous cells with variable nuclear chromatin, an irregular nuclear shape, 1 or more nucleoli, a variable amount of cytoplasm, and variable basophilia

ALL-L3: Large, homogeneous cells with fine, stippled chromatin; regular nuclei; prominent nucleoli; and abundant, deeply basophilic cytoplasm. The most distinguishing feature is prominent cytoplasmic vacuolation

### Genome-wide methylation profiling identifies specific methylation signatures in leukemia

**Fig. 1:**
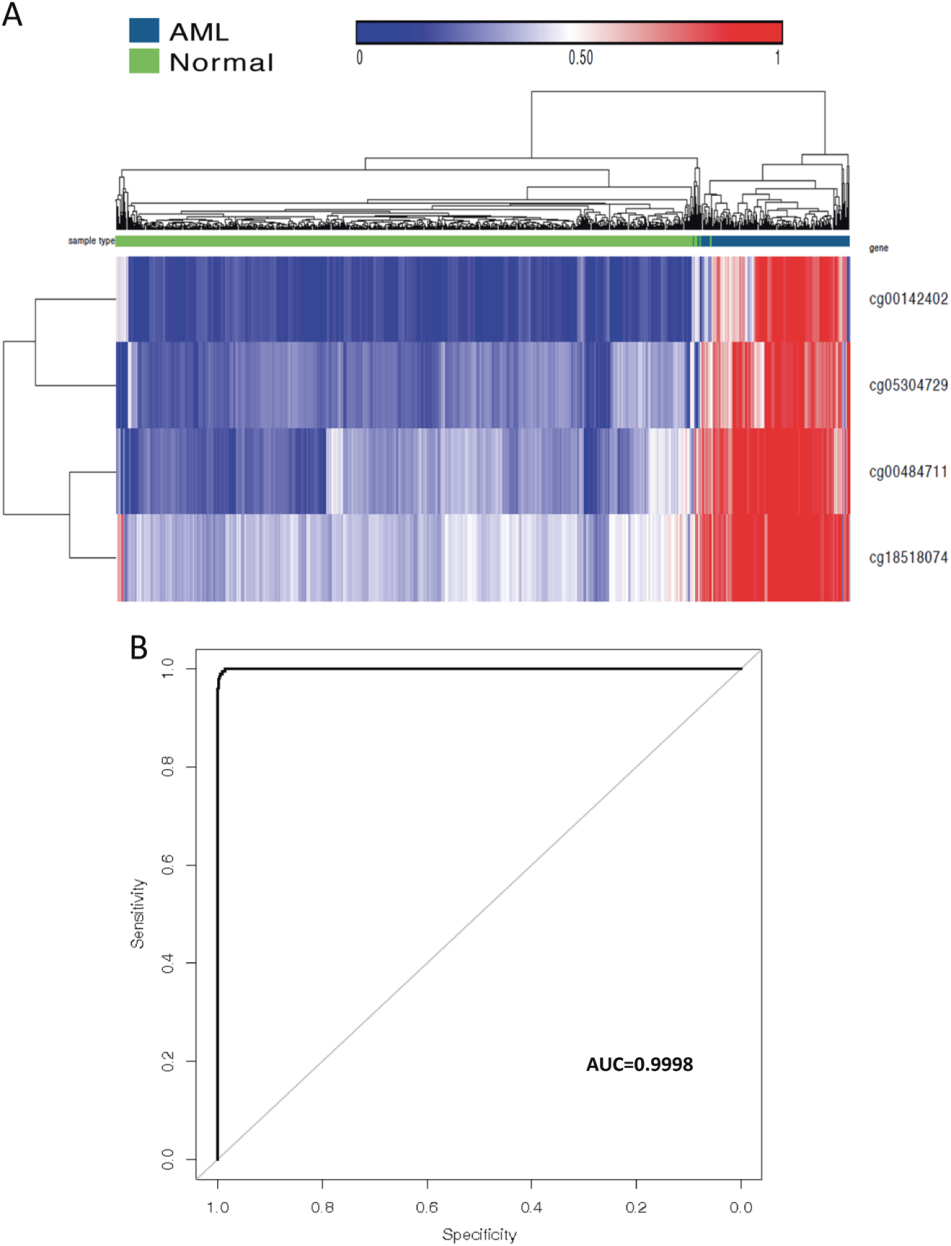
Methylation profile can differentiate AML blood and normal blood using 4 markers. **a** Unsupervised hierarchical clustering and the heat map associated with the methylation profile (according to the color scale shown) in AML blood vs normal blood. **b** The accuracy of predicting AML as assessed by the ROC curve.

We randomly split the TCGA AML samples, Chinese ALL samples, and normal blood samples of healthy controls into training and validation data sets at a 70/30 ratio using *R*. We then compared methylation differences between the TCGA AML samples and normal blood samples and between the Chinese ALL samples and normal blood samples in the training data sets using the nearest shrunken centroids method.^19^ Two sets of CpG sites were then identified and used to differentiate the TCGA AML samples from normal blood samples and the Chinese ALL samples from normal blood samples in the validation data sets. This method of random splitting was repeated 20 times. Tables 2A, 2B, 3A, 3B shows confusion tables describing the performance of these classifiers in differentiating AML and ALL samples from normal blood samples on one of the 20 training and validation data sets. The 20 sets of CpG sites identified through AML-normal comparison revealed four common CpG sites. These four CpG sites were plotted in an unsupervised fashion in AML versus normal blood samples (Fig. 1a). The accuracy of using these four CpG sites for predicting AML leukemia was assessed by the ROC curve (Fig. 1b), which had an AUC of 0.9998.

**Table 2:** Confusion table of training cohort. (A) Confusion table of AML and normal blood; (B) Confusion table of ALL and normal blood; (C) Confusion table of AML and ALL.

A			
Training cohort	AML	Normal blood	
AML	134	1	
Normal blood	135	526	Totals
Totals	134	527	662
Correct	134	526	660
False positive	0	1	1
False negative	1	0	1
Specificity (%)		99.8	99.8
Sensitivity (%)	99.3		99.8

B			
Training cohort	ALL	Normal blood	
ALL	94	0	
Normal blood	1	527	Totals
Totals	95	527	662
Correct	94	527	621
False positive	0	0	0
False negative	1	0	1
Specificity (%)		100	100
Sensitivity (%)	98.9		99.8

C			
Training cohort	AML	ALL	
AML	135	0	
ALL	0	95	Totals
Totals	135	95	230
Correct	135	95	230
False positive	0	0	0
False negative	0	0	0
Specificity (%)		100	100
Sensitivity (%)	100		100

**Table 3:** Confusion table of validation cohort. (A) Confusion table of AML and normal blood; (B) Confusion table of ALL and normal blood; (C) Confusion table of AML and ALL.

A			
Validation cohort	AML	Normal blood	
AML	59	6	
Normal blood	0	221	Totals
Totals	59	227	286
Correct	59	221	280
False positive	0	6	6
False negative	0	0	0
Specificity (%)		97.4	97.9
Sensitivity (%)	100		100

B			
Validation cohort	ALL	Normal blood	
ALL	41	0	
Normal blood	0	227	Totals
Totals	41	227	268
Correct	41	227	268
False positive	0	0	0
False negative	0	0	0
Specificity (%)		100	100
Sensitivity (%)	100		100

C			
Validation cohort	AML	ALL	
AML	59	0	
ALL	0	41	Totals
Totals	59	41	100
Correct	59	41	100
False positive	0	0	0
False negative	0	0	0
Specificity (%)		100	100
Sensitivity (%)	100		100

**Fig. 2:**
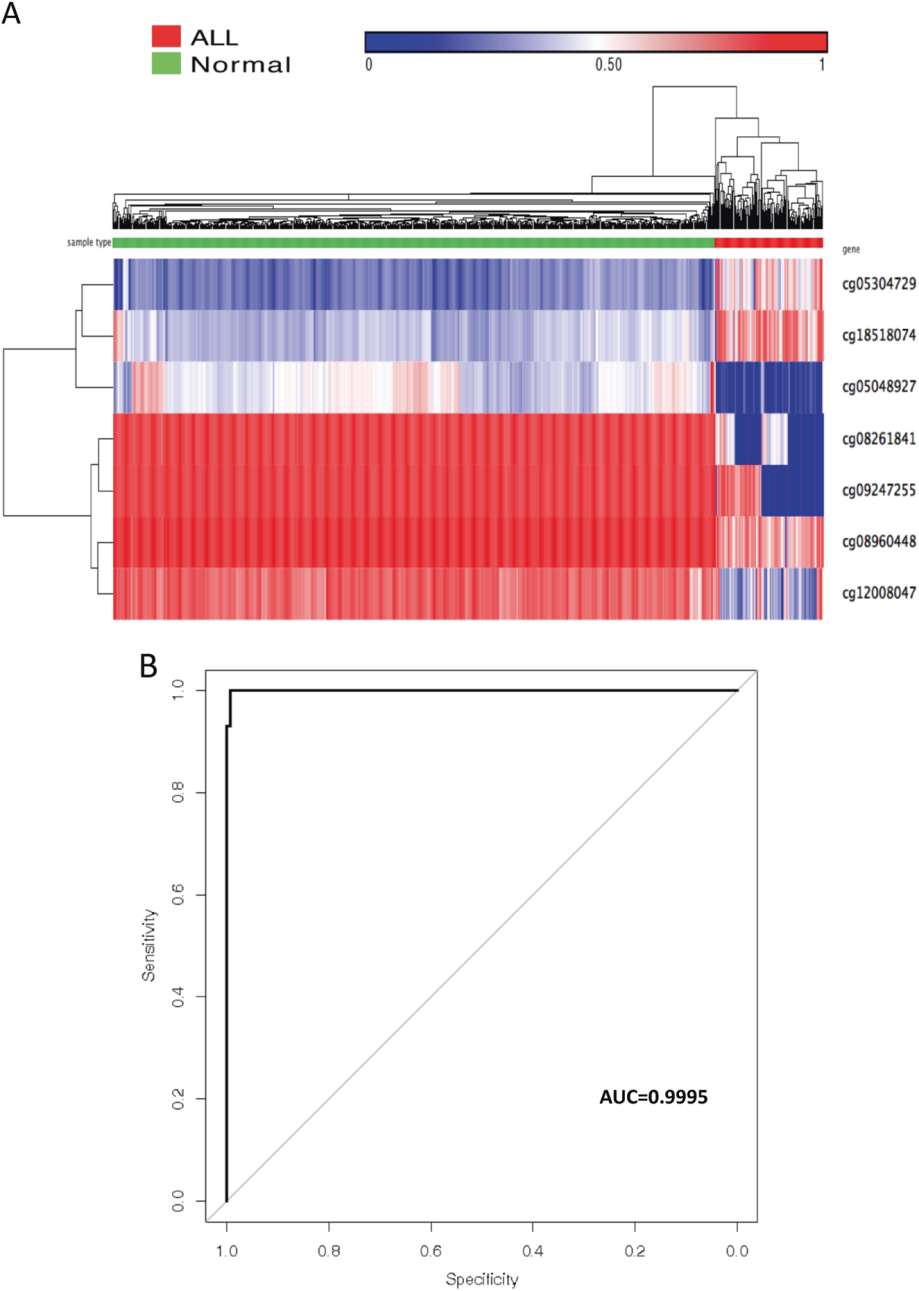
Methylation profile can differentiate ALL blood and normal blood using 7 markers. **a** Unsupervised hierarchical clustering and the heat maps associated with the methylation profile (according to the color scale shown) in ALL blood versus normal blood samples. **b** The accuracy of predicting ALL as assessed by the ROC curve.

Similarly, we identified seven common CpG sites through the ALL-normal comparison (Fig. 2a). The accuracy of using these seven CpG sites for predicting ALL leukemia was assessed by the ROC curve (Fig. 2b), which had an AUC of 0.9995. It is worth noting that two common CpG sites (cg05304729 and cg18518074) appeared both in the AML-normal comparison and in the ALL-normal comparison (Figs. 1a, 2a). Taken together, these data demonstrated that differential methylation of CpG sites was able to distinguish the blood of particular leukemia types from normal blood with high specificity and sensitivity (Figs. 1b, 2b). Overall, these results demonstrate the robust nature of these methylation patterns in identifying the presence of a particular type of leukemia.

### Methylation profiles can distinguish between different leukemia

**Fig. 3:**
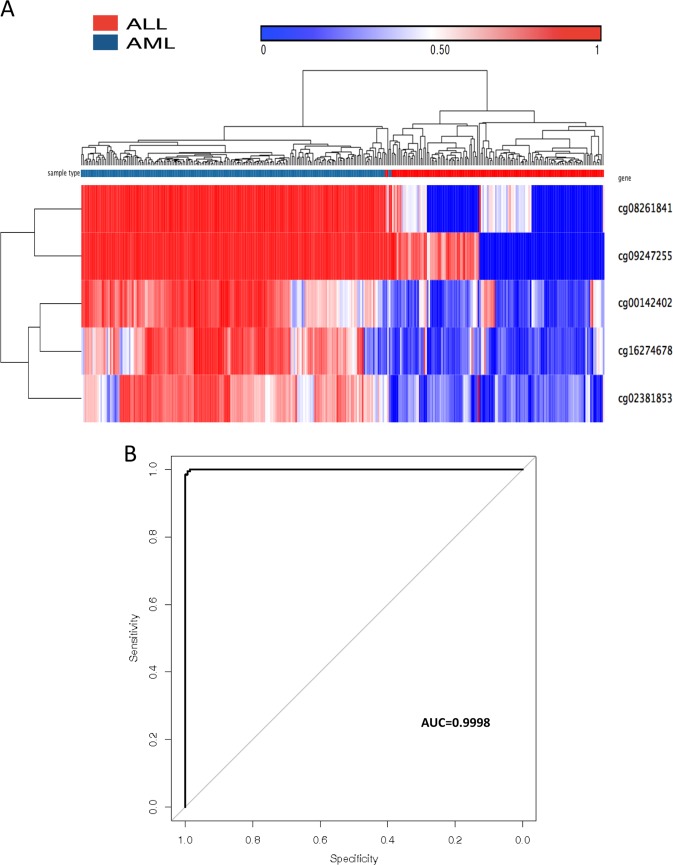
Methylation profile can differentiate subtypes of leukemia using 5 markers. **a** Unsupervised hierarchical clustering and the heatmap with the methylation profile (according to the color scale shown) in ALL versus AML samples. **b** The accuracy of predicting AML and ALL as assessed by the ROC curve.

**Fig. 4:**
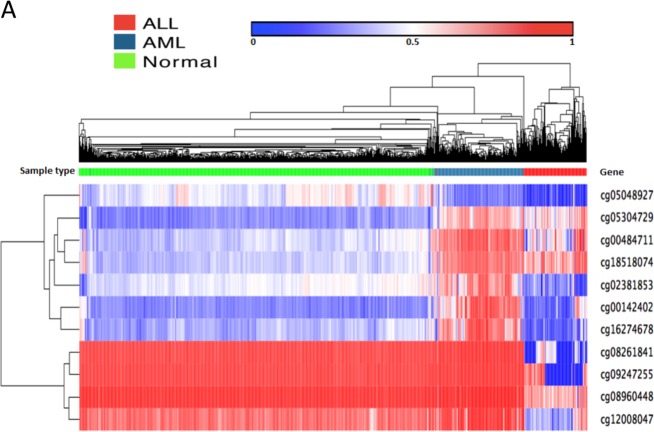
Using 11 markers, the methylation profile can differentiate the leukemia subtype and normal blood. Unsupervised hierarchical clustering and the heatmap associated with ALL, AML, and normal blood.

We have shown the ability of our method to distinguish between the blood of particular types of leukemia and normal blood samples. We then investigated whether our algorithm was able to distinguish different types of leukemic cancers (ALL and AML) arising from bone marrow. We identified five CpG sites that could be used to differentiate the TCGA AML samples from our Chinese ALL samples (Fig. 3a) and generated confusion tables (Tables 2C, 3C) describing the performance of our classifiers on one of 20 training and validation data sets consisting of the TCGA AML samples and the Chinese ALL cohort samples used in Tables 2A, 2B, 3A, 3B. It is worth noting that among these five CpG sites, one (cg00142402) was also identified in the AML and normal comparison, and two (cg08261841 and cg09247255) were also identified in the ALL and normal comparison. The accuracy of using these five CpG sites for differentiating between AML and ALL can be assessed by the ROC curve (Fig. 3b), which had an AUC of 0.9998. Together, these results demonstrate the efficacy of using methylation patterns for the accurate diagnosis of a cancer histological subtype. The 11 unique CpG sites that could differentiate among TCGA AML, Chinese ALL and normal blood samples are plotted in an unsupervised fashion in Fig. 4.

### Methylation profiles predict prognosis and survival rates

**Fig. 5:**
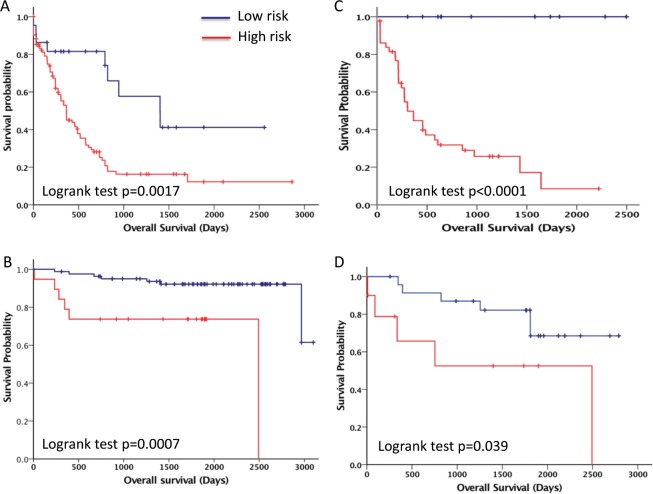
Methylation markers can predict the five-year overall survival of patients. **a** AML training set (*n* = 125); **b** AML validation set (*n* = 55); **c** ALL training set (*n* = 55); and **d** ALL validation set (*n* = 34).

We investigated the effect of methylation markers on the survival rate of each leukemia subtype (AML and ALL) based on a semisupervised method.^20^ Specifically, for each leukemia subtype, the CpG sites in the training data were ranked based on their Cox scores. Thirty-nine CpG sites whose Cox scores exceeded 2.197 (corresponding to the 96th percentile of the AML Cox scores) and 93 CpG sites whose Cox scores exceeded 3.215 (corresponding to the 92nd percentile of the ALL Cox scores) were selected, and their methylation profiles were used to classify 125 AML patients and 102 ALL patients, respectively, into “good survival” or “bad survival” by the 2-means clustering method. The resulting two subgroups for each leukemia subtype (AML and ALL) showed the most significant difference with respect to survival, and from these subgroups, we also obtained two optimal classification models: one contained 20 methylation signatures for the AML subtype, and one contained 23 methylation signatures for the ALL subtype (see the methods section). These two classifiers were then used to classify the 55 AML patients and the 34 ALL patients in the validation cohort. Individual patient survival data were plotted using a Kaplan–Meier curve (Fig. 5). A similar result was also observed in the whole cohort (Fig. S1). These methylation signatures were able to predict highly significant differences in the survival of patients with ALL and AML.

## Discussion

Tumor-specific methylation patterns have been widely studied for their potential in cancer diagnosis and prognosis.^21–23^ Due to the high cost of whole-methylome sequencing, targeted specific methylation positions have been more commonly surveyed in tumor methylation marker discovery screening. For example, our previous work on hepatocellular carcinoma utilized a 401 padlock probe set and found ten CpG markers for diagnosis and eight CpG markers for prognosis.^16^ In this study, we designed a padlock-based bisulfate sequencing method using data from the TCGA database. We demonstrated that differential methylation of CpG sites was able to distinguish the blood from a particular leukemia type from normal blood with high specificity and sensitivity (Tables 2, 3). We also demonstrated our ability to distinguish histologic subtypes of leukemia (ALL and AML) derived from the same tissue in the bone marrow (Tables 2, 3). Furthermore, we showed that methylation patterns can predict survival in ALL or AML patients and revealed subsets of patients with either a significant positive or negative prognosis. This finding raises the possibility that methylation may help to identify relatively benign or aggressive tumors and may aid in decision-making regarding the selection of more or less aggressive treatment and monitoring. DNA methylation patterns likely represent common pathways of carcinogenesis and may be more reproducibly altered in cancers, potentially allowing more robust diagnosis and prognostication than somatic mutations. Indeed, methylation patterns may capture the biological state of a cell more accurately than histopathology or somatic mutations alone.

Our data have significant implications for improving the diagnostic yield for biopsies from patients whose bone marrow biopsy results are inconclusive, which often occurs due to artificial tissue distortion. These results may further be helpful to identify leukemic subtypes in cases in which the tissue yield or quality is inadequate for histology to make an accurate diagnosis, as histology requires preservation of the tissue architecture.^24^ In fact, it was often a dilemma with biopsies to balance between specimen yield and quality and discomfort or potential complications such as hemorrhage.^25,26^ Moreover, bone marrow pathological examinations are often relatively time-consuming, and diagnosis based on morphology can be inconclusive or inconsistent depending on the personal experience of pathologists. In contrast, DNA methylation analysis requires only a small amount of tissue to obtain adequate DNA, thus potentially allowing the use of lower quality biopsies. The ability to identify histologic subtypes for these cancers within the bone marrow has important implications because different cancers confer different prognoses and require distinct treatment plans; diagnostic failure or uncertainty may lead to less favorable outcomes and survival.

It may not be surprising that DNA methylation patterns have such differentiating abilities in distinguishing between the blood of subtypes of leukemia and normal blood. It is known that many genes involved in the methylation machinery are mutated in leukemia (TET2, TPMT, and DNMT3A),^27–31^ therefore leading to significant alteration in methylation patterns.

Recently, a number of prognostic factors have been proposed for AML and ALL, such as clinical features, immunophenotype, and cytogenetic and molecular characteristics.^19,32–34^ The identification of prognostic factors, an improved stratification of risk groups and survival analyses have made it possible to identify the presence of the disease and evaluate treatment outcomes.^35,36^ However, the clinical utilities of gene mutation analysis, gene expression profiling, and microRNA analysis remain uncertain at this time. Flow cytometry also provides a direct assessment of surface antigen expression profiles on leukemic cells,^37^ facilitating the rational and individualized selection of targeted immunotherapy strategies. Several advances in flow cytometry, including the availability of new monoclonal antibodies, improved gating strategies, and multiparameter analytic techniques, have all dramatically improved its utility in the diagnosis and classification of leukemia. However, morphologic and differentiation-based classifications of leukemia are limited by their prognostic value, as well as the available monoclonal antibodies.

In this study, we also applied methylation profiling and machine learning analysis to the survival data of ALL and AML patients. Interestingly, we were able to separate each leukemic type we examined into distinct groups with better or worse survival outcomes. These results also support the idea that methylation patterns may offer a more accurate picture of the biological state of a cancer than histology and IHC alone or even somatic mutation analysis. However, we expect that a combination of all of these methods is most likely to offer the most complete and useful information for treating patients with leukemia. One known prognostic factor is the origin from which progenitor leukemic cancer cells are derived from during hematopoiesis, as leukemic cells from more differentiated progenitors carry a better prognosis. Therefore, it would be interesting to see if leukemic patients with better prognosis/survival based on a methylation signature have the characteristics of a more differentiated disease.

Additionally, the blood can be taken from the patients at any time during the course of therapy, which facilitates the use of the methylation profile for dynamic monitoring of the epigenetic changes of leukemic cells instead of repetitive bone marrow biopsies. It also allows for the detection of minimal residual disease and the prediction of the risk of relapse.

In summary, we identified a CpG methylation panel for the diagnosis and prognosis of common leukemia with high sensitivity and specificity. Our results support the potential clinical utility of DNA methylation signatures to distinguish leukemia types and to predict prognosis and outcomes.

### Key points

ALL and AML have specific DNA methylation signatures that are associated with cancer-related gene expression regulation.

DNA methylation markers can differentiate AML from ALL.

DNA methylation markers can provide prognosis and survival assessment for AML and ALL patients.

## Methods

### Patient data

Patient data of the AML training and validation cohorts were obtained from The Cancer Genome Atlas (TCGA). Patient characteristics are summarized in Table 1. Complete clinical, molecular, and histopathological data sets are available at the TCGA website: https://tcga-data.nci.nih.gov/tcga/. Individual institutions that contributed samples coordinated the consent process and obtained informed written consent from each patient in accordance with their respective institutional review boards.

The second independent (Chinese) ALL cohort consisted of patients from Guangzhou Women and Children’s Medical Center, China, and patient characteristics are summarized in Table 1. This project was approved by the IRB of Guangzhou Women and Children’s Medical Center. Informed consent was obtained from all patients. Tumor and normal tissues were obtained as clinically indicated for patient care and were retained for this study with patients’ informed consent.

### Data sources

DNA methylation data were obtained from both the TCGA analysis of 485,000 sites generated using the Infinium 450K Methylation Array and the following GSE data set: GSE40279. Methylation profiles for AML cancer types and their corresponding normal blood were analyzed. IDAT format files of the methylation data were generated containing the ratio values of each scanned bead. Using the minfi package from Bioconductor, these data files were converted into a score, referred to as a beta value. Methylation data of the Chinese cohort were obtained by padlock-based bisulfate sequencing of a pancancer marker set and were analyzed as described below.

### Generating methylation markers enriched in cancer

We selected 729 previously reported CpG markers that showed differential methylation values in many cancer types when compared to the corresponding normal tissues.^18^

### Classifying samples

For classifying the ALL, AML, and normal blood samples, we applied a supervised learning technique, the “nearest shrunken centroids” procedure of Tibshirani et al.^38^, which is implemented in the package PAM.^39^ Specifically, we first mixed the TCGA AML samples, Chinese ALL samples and normal blood samples. Seventy percent of these combined samples were put into the training set, and thirty percent were put into the validation set. We then performed the PAM procedure with 10-fold cross-validation on the training data set and obtained robust classifiers for each AML-normal, ALL-normal, and AML-ALL comparison. These classifiers were then used to classify the validation data. This leave-group-out cross-validation was repeated 20 times.

To predict survival in each leukemia subtype (AML and ALL), we applied a semisupervised method proposed by Bair and Tibshirani.^20^ Specifically, the patient cohorts were randomly divided into a training set (*n* = 125 for AML and *n* = 102 for ALL) and a validation set (*n* = 55 for AML and *n* = 34 for ALL). For each CpG site, we fit a univariate Cox proportional hazard regression model with survival outcome and methylation value as predictors using the training data set. These CpG sites were then ranked based on their Cox scores. For a given Cox score cutoff, we obtained a list of CpG sites whose Cox scores exceed the cutoff. Then, we performed 2-means clustering on the training patients and obtained two subgroups for each leukemia subtype. We then conducted log-rank tests on the survival of these two subgroups for each leukemia subtype and applied the nearest shrunken centroids model with cross validation to train a classification model. We examined 100 equally spaced Cox scores between the 90th percentiles of the Cox scores and the maximum of the Cox scores. The optimal Cox score cutoff was chosen such that the resulting two subgroups for each leukemia subtype differed most significantly with respect to survival, and the resulting classification model had the smallest cross validation error. We then used the trained classification models, one for AML and one for ALL, to predict the subgroup to which each patient in the AML and ALL validation sets belonged. The 20 methylation signatures for survival in AML and the 23 methylation signatures for survival in ALL are listed below.

**AML**: cg01336231, cg01413582, cg01509330, cg02264990, cg02329430, cg02858512, cg03297901, cg03556653, cg04596071, cg05038216, cg06034933, cg08098128, cg13066703, cg17757602, cg18869709, cg19966212, cg20300129, cg23193870, cg23680451, and cg25145765.

**ALL**: cg01628067, cg03001333, cg04984818, cg05145233, cg05304729, cg05956452, cg06261066, cg09157302, cg14608384, cg15289427, cg15608301, cg15707093, cg16266227, cg18869709, cg19470372, cg19864130, cg20686234, cg21913319, cg24720672, cg24747122, cg24983367, cg26584619, and cg27178401.

In our analysis, we observed four potential types of classification errors.

1. False negative; e.g., ALL blood was identified as normal blood.
2. False positive; e.g., normal blood was identified as ALL or AML blood.
3. Correct sample, incorrect leukemia type; e.g., ALL blood was identified as AML blood.

### Tumor DNA extraction

Genomic DNA extraction from normal blood or ALL bone marrow cancer samples was performed with the QIAamp DNA Mini Kit (Qiagen) according to the manufacturer’s recommendations. DNA was stored at −20 °C and analyzed within 1 week of preparation.

### Bisulfite conversion of genomic DNA

Up to 1 µg of genomic DNA was converted to bis-DNA using an EZ DNA Methylation-Lightning™ Kit (Zymo Research) according to the manufacturer’s protocol. The resulting bis-DNA had a size distribution of ~200–3000 bp, with a peak around ~500–1000 bp. The efficiency of bisulfite conversion was >99.8%, as verified by deep sequencing of bis-DNA and analyzing the ratio of the C to T conversion of CH (non-CG) dinucleotides.

### Determination of DNA methylation levels of the ALL cohort by deep sequencing of bis-DNA captured with molecular-inversion (padlock) probes

A total of 729 CpG markers whose methylation levels significantly differed in any of the comparisons between leukemic and normal tissue were used to design padlock probes for sequencing. Padlock-capture of bis-DNA was based on published techniques and protocols with modifications.^17,40,41^

#### Determination of DNA methylation levels by deep sequencing of bis-DNA captured with molecular inversion (padlock) probes

Padlock probes were designed to capture regions containing the CpG markers whose methylation levels significantly differed in comparison between leukemic and normal blood. Padlock-capture of bis-DNA was based on published techniques and protocols with modifications.^40,41^

#### Probe design and synthesis

Padlock probes were designed using the ppDesigner software. The average length of the captured region was 100 bp, with the CpG marker located in the central portion of the captured region.

#### Bis-DNA capture

For this analysis, 100 ng of bisulfite-converted DNA was annealed to padlock probes in 20 µl reactions containing 1× Ampligase buffer (Epicenter). To anneal probes to DNA, 30 s of denaturation at 95 °C was followed by a slow cooling to 55 °C. To fill gaps between annealed arms, the following mixture was added to each reaction: Pfu polymerase (Agilent), 0.5 U of Ampligase (Epicenter) and 250 pmol of each dNTP in 1× Ampligase buffer. After 5 h of incubation at 55 °C, the reactions were denatured for 2 min at 94 °C and snap-cooled on ice. Exonuclease mix (20 U of ExoI and 100 U of ExoIII, both from Epicenter) was added, and single-stranded DNA degradation was carried out at 37 °C for 2 h, followed by enzyme inactivation for 2 min at 94 °C.

Circular products of the above CpG site-specific capture were amplified by PCR with concomitant barcoding of separate samples. Amplification was carried out using primers specific to linker DNA within the padlock probes, one of which contained specific 6 bp barcodes. Both primers contained Illumina next-generation sequencing adapter sequences. PCR of the captured DNA was performed using Phusion Flash Master Mix (Thermo) and a 200 nM final concentration of primers under the following cycle conditions: 10 s @ 98 °C; 8 cycles of 1 s @ 98 °C, 5 s @ 58 °C, and 10 s @ 72 °C; 25 cycles of 1 s @ 98 °C and 15 s @ 72 °C; and 60 s @ 72 °C. PCRs were mixed, and the resulting library was size selected to include effective captures (~230 bp) and exclude “empty” captures (~150 bp) using Agencourt AMPure XP beads (Beckman Coulter). The purity of the libraries was verified by PCR using Illumina flowcell adapter primers (P5 and P7), and the concentrations were determined using the Qubit dsDNA HS assay (Thermo Fisher). Libraries we sequenced using the MiSeq and HiSeq2500 systems (Illumina).

#### Sequencing data analysis

The sequencing reads were mapped using the software tool bisReadMapper with some modifications. First, UMI were extracted from each sequencing read and appended to read headers within the FASTQ files using a custom script generously provided by D.D. Reads were on-the-fly converted as if all Cs were nonmethylated and mapped to in-silico converted DNA strands of the human genome, also as if all Cs were nonmethylated, using Bowtie 2.^42^ Original reads were merged and filtered for single UMI, i.e., reads carrying the same UMI were discarded, leaving a single one. Methylation frequencies were extracted for all CpG markers for which padlock probes were designed. Markers with less than 20 reads in any sample were excluded from analysis. This resulted in ~600 CpG markers for which the methylation level was determined with an accuracy of 5% or more.

## Supplementary information

Supplementary Figure 1
