# Supplementary material for: DNA methylation markers in the diagnosis and prognosis of common leukemias
Source: Signal Transduct Target Ther. 2020 Jan 10;5:3. doi: 10.1038/s41392-019-0090-5 (PMC6959291; doi:10.1038/s41392-019-0090-5)

Supplementary Materials for

DNA methylation markers in diagnosis and prognosis of common leukemia

Hua Jiang^#,*1^, Zhiying Ou^#1^, Yingyi He^#1^, Meixing Yu^#1^, Shaoqing Wu^1^, Gen Li^1^, Jie Zhu^1^, Ru Zhang^1^, Jiayi Wang^1^, Lianghong Zheng^2^, Xiaohong Zhang^1^, Wenge Hao^1^, Liya He^1^, Xiaoqiong Gu^1^, Qingli Quan^1^, Edward Zhang^1^, Huiyan Luo^3^, Wei Wei^3^, Zhihuan Li^2^, Guangxi Zang^2^, Charlotte Zhang^1^, Tina Poon^1^, Daniel Zhang^1^, Ian Ziyar^2^, Run-ze Zhang^2^, Oulan Li^2^, Linhai Cheng^2^, Taylor Shimizu^2^, Xinping Cui^4^, Jian-kang Zhu^5^, Xin Sun^*1^, and Kang Zhang^*6^

Correspondence to: [Jiang.Hua33@yeah.net, doctorsunxin@hotmail.com, or kang.zhang@gmail.com](mailto:xxxxx@xxxx.xxx)

**This PDF file includes:**

Figures. S1

Figure. S1. Methylation markers can predict five-year overall survival of the whole patients cohort with: A. AML (n=180); B. ALL (n=136).


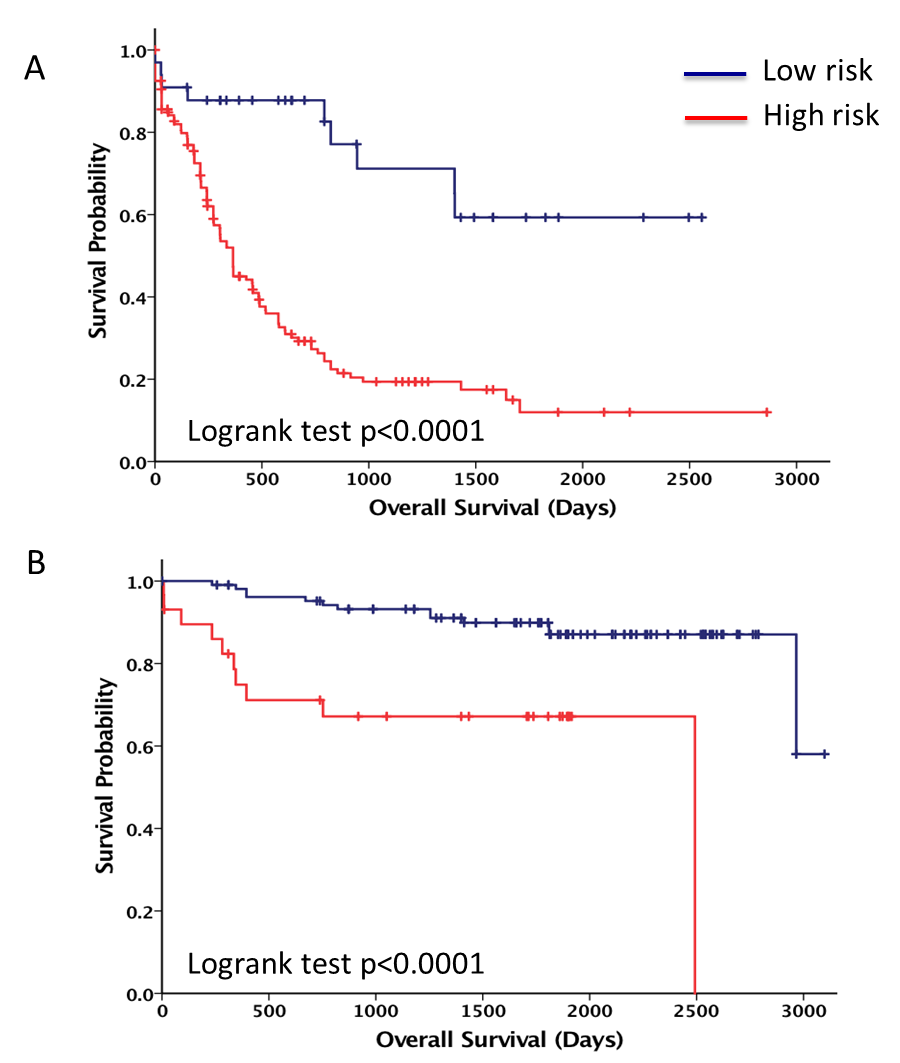

Supplement: Supplementary file 1 — Supplementary Figure 1 [file 41392_2019_90_MOESM1_ESM.docx]
